# Radiolabeling, biodistribution and gamma scintigraphy of noscapine hydrochloride in normal and polycystic ovary induced rats

**DOI:** 10.1186/1757-2215-3-10

**Published:** 2010-04-27

**Authors:** Anjali Priyadarshani, Krishna Chuttani, Gaurav Mittal, Aseem Bhatnagar

**Affiliations:** 1 Department of Zoology, K M College, University of Delhi, Delhi-110 007, India; 2 Institute of Nuclear Medicine and Allied Sciences, Brig. S. K. Mazumdar Road, Delhi-110 054, India

## Abstract

**Background:**

Noscapine, an alkaloid from *Papaver somniferum*, widely used as an antitussive, is being clinically studied in the treatment of polycystic ovary syndrome (PCOS) and a few other cancers primarily because of its anti-angiogenesis properties. With the advent of diverse application of noscapine, we sought to determine whether the radiolabeling method can be useful in studying uptake and kinetics of the molecule in-vivo. Specific objectives of this study were to radiolabel noscapine with Technetium-99m (Tc-99m), to determine its organ biodistribution in rat model and study its uptake kinetics in PCOS model.

**Methods:**

A method for radiolabeling noscapine with Tc-99m was standardized using stannous reduction method and its in vitro and in vivo stability parameters were studied. The radiopharmaceutical was also evaluated for blood kinetics and biodistribution profile. An animal model of PCOS was created by using antiprogesterone RU486 and uptake of ^99m^Tc-noscapine in normal and PCOS ovaries was compared using gamma scintigraphy.

**Results:**

Noscapine hydrochloride was successfully radiolabeled with Tc-99m with high labeling efficiency and in vitro stability. Most of the blood clearance of the drug (80%) took place in first hour after intravascular injection with maximum accumulation being observed in liver, spleen, kidney followed by the ovary. At 4 hours post injection, radiolabeled complex accumulation doubled in PCOS ovaries in rats (0.9 ± 0.03% ID/whole organ) compared to normal cyclic rats (0.53 ± 0.01% ID/whole organ). This observation was further strengthened by scintigraphic images of rats taken at different time intervals (1 h, 2 h, 4 h, and 24 h) where SPECT images suggested discrete accumulation in the PCOS ovaries.

**Conclusion:**

Through our study we report direct radiolabeling of noscapine and its biodistribution in various organs and specific uptake in PCOS that may show its utility for imaging ovarian pathology. The increased ovarian uptake in PCOS may be related to its receptor binding suggesting possible role of ^99m^Tc-noscapine in PCOS diagnostics and therapeutics.

## Background

Noscapine, a phthalideisoquinoline alkaloid has long been used as a cough suppressant in humans and in experimental animals[1,2]. Unlike other opioids, noscapine lacks sedative, euphoric, and respiratory depressant properties [3] and is free from serious toxic effects in doses up to 100 times the antitussive dose [4]. Recently, anticancer properties of noscapine have been reported and it has been shown that noscapine interacts with α tubulin resulting in apoptosis in cancerous cells both *in vitro *and *in vivo *[5-8]. Moreover, noscapine is also shown to reduce neoangiogenesis resulting in reduced cell turnover. Its role in tumor and tumor-like conditions is therefore being investigated with great interest [9,10].

Although animal studies have shown the therapeutic potential of noscapine in inhibiting cancer progression in animal models [10,11], there has been no study to ascertain whether noscapine can be used in the diagnosis of developing tumors, including those inflicting the ovaries. We were therefore interested in exploring the possibility of using nuclear medicine techniques, including gamma scintigraphy for detecting ovarian dysfunctions using noscapine. Polycystic ovarian syndrome (PCOS) was chosen as the model system to study noscapine uptake because of its easy inducibility [12]. Though pharmacotherapies like metformin, clomiphene citrate and flutamide have been used for the treatment of PCOS, serious side effects with long treatment schedule makes them unapproachable [13-16]. Consequently, there is an urgent need for better drugs that can target the core of PCOS, hypothalamus-pituitary-ovarian (HPO) axis and normalize the broad spectrum of PCOS anomalies with minimal side effects [17]. Keeping the drawbacks of existing therapeutic modalities for the syndrome in mind, the present investigation also aimed to give important leads to investigators for developing noscapine as a novel alternative for treatment of various ovarian dysfunctions, including PCOS.

Since the plasma half-life of noscapine is 2.5 h to 4.5 h[18,19] it is theoretically quite compatible with the physical half-life of 6 hrs of Technetium-99m (Tc-99m). Moreover, noscapine is grouped as part of the benzylisoquinolines, and possesses certain electron rich sites such as methoxy side chain, N-methyl group, carbonyl, lactonyl, dioxide site that makes it available for binding to radioisotopes such as Tc-99m [20]. Keeping in view the inherent properties of Tc-99m, attempts were made to radiolabel noscapine with Tc-99m. We then sought to determine various factors influencing the radiolabeling process and *in vitro *stability of the labeled complex. Subsequently blood kinetics in rabbits; and tissue distribution and gamma scintigraphy studies of ^99m^Tc-noscapine were performed in female rats. Furthermore, levels of labeled noscapine in ovary of healthy rats were compared with the rats induced with precancerous conditions of polycystic ovary syndrome (PCOS) wherein the theca cell turnover is significantly more than the healthy controls [21]. The objective was to generate organ distribution data with respect to noscapine using nuclear medicine techniques and to ascertain whether radiolabeled noscapine can have a diagnostic application in ovarian dysfunction, with particular reference to PCOS.

## Methods

### Drugs/Chemicals

Noscapine hydrochloride and Antiprogesterone RU486, 11β-(4-dimethyl amino phenyl)-1 β-hydroxy-17α-(1-propenyl)-oestra-4, 9-diene-3-one were procured from Sigma Chemical Co., St. Louis, MO, while Tc-99m was eluted from ^99^Mo by methyl ethyl ketone extraction and provided by BRIT, BARC, India. All the chemicals used in this study were of analytical grade.

### Animals

Female New Zealand rabbits weighing approximately 2.25 ± 2 kg and adult female Wister rats (aged 12-14 weeks, body weight 200 ± 4.5 g) were housed in animal house facility at Institute of nuclear medicine and allied sciences, under controlled light (12 h light: 12 h dark) and temperature (22-24°C) conditions. The animals were provided water and their respective chow. Animal handling and experimentation was carried out as per the guidelines of the institutional animal ethics committee.

Radiolabeling and its subsequent quality control parameters, including radiochemical purity, in vitro and in vivo stability, blood kinetics and biodistribution studies were broadly determined as per established nuclear medicine procedures [22-27]. However, procedural details with respect to noscapine have been given in the subsequent sections.

### Radiolabeling of Noscapine with Tc-99m

^99m^Tc-noscapine was prepared by dissolving 500 μg of noscapine hydrochloride in 1 ml of distilled water followed by the addition of 50 μg of SnCl_2_.2H_2_O, the pH being adjusted to 6.5. The contents were filtered through 0.22 μm membrane filter (Millipore Corporation, Bedford, MA USA) into a sterile vial. Approximately 55-60 MBq Tc-99m was added to the contents, mixed and incubated for 5-10 min. The percent radiolabel was determined by using instant thin layer chromatography (ITLC) by the method previously reported from our lab [27].

### Effect of concentration of stannous chloride and pH on the labeling efficiency

To examine the effect of varying concentration of SnCl_2_.2H_2_O on labeling efficiency, amount of SnCl_2_.2H_2_O was varied from 10 to 400 μg keeping the pH constant at 6.5. In another experiment, the amount of stannous chloride dihydrate was kept constant (50 μg) while the pH was varied from 4 to 7 by adding 0.5 M NaHCO_3_. The experiment was performed in triplicate and labeling yield was measured using 100% acetone as the mobile phase. Percentage of colloids was detected by pyridine: acetic acid: water (3:5:1.5 v/v) as the mobile phase.

### Radiochemical purity

The radiochemical purity of Tc-99m with noscapine was estimated by instant thin layer chromatography (ITLC) using silica gel coated fibre sheets (Gelman Sciences. Inc., Ann Arbor, MI USA). ITLC was performed using 100% acetone and 0.9% saline as the mobile phase. A measured amount of 2-3 μl of the radiolabeled complex was applied at a point 1 cm from one end of an ITLC-SG strip and allowed to run for approximately 10 cm. Amount of reduced/hydrolyzed Tc-99m was determined using pyridine: acetic acid: water (3:5:1.5 v/v) as mobile phase and ITLC as the stationary phase.

### In vitro and in vivo stability

For determining *in vitro *stability of the radiolabel, 450 μl each of 0.9% saline and rat serum were mixed separately with 50 μl of the radiolabeled complex and incubated at 37°C. Aliquots made were subjected to ITLC at different time intervals in 100% acetone. *In vivo *stability was assessed by administering 300 μl of ^99m^Tc-noscapine (18.5 MBq) to New Zealand albino rabbits through the ear vein and withdrawing blood samples at different time intervals which were then subjected to ITLC.

### Blood kinetics

Blood clearance of the labeled noscapine was studied in healthy female rabbits weighing 2.25 ± 2 kg. 18.5 MBq activity of the radiolabeled conjugate was injected intravenously through the dorsal ear vein of the rabbit. Blood was drawn at different time intervals from the other ear using sterile syringes, and its radioactivity was measured by taking 7% of the body weight as the total blood volume. The data was expressed as percent administered dose present in whole body blood at each time interval.

### Biodistribution of radiocomplexed drug

Female rats weighing 200 ± 4 g were selected for evaluating localization of the labeled complex. ^99m^Tc-noscapine (80KBq) was administered through the tail vein of each rat. Groups of 3 rats per time point were used in the study. The organ distribution studies of labeled noscapine were evaluated after 0.25 h, 1 h, 2 h, 4 h, and 24 h post injection. At these time intervals, blood was collected by cardiac puncture and the animals were humanely sacrificed. Subsequently, tissues (heart, brain, ovary, lung, spleen, kidney, stomach. intestine and bone) were removed, washed with normal saline, made free from adhering tissues and weighed. The radioactivity in each organ was counted in gamma counter and expressed as percent injected dose per whole organ [27].

### Establishment of animal model for polycystic ovary syndrome (PCOS)

The laboratory rat has been frequently used as an animal model to study persistent estrus associated with PCOS condition. PCOS animal model was established using antiprogestin, mifepristone, with slight modifications in the method employed by Sanchez-Criado [28,29]. Rats weighing 200 ± 4 g showing at least three consecutive 4-5 day estrous cycles were orally administered RU486 (20 mg/Kg b wt./day) in olive oil daily for consecutive 13 days, starting on the day 1 of the estrous cycle. Polycystic ovary syndrome in rat models represents the induction of polycystic ovaries associated with persistent vaginal cornification (PVC), which signifies chronic anovulation. Therefore, the animals were checked for vaginal cornification in vaginal smears microscopically and changes in reproductive cycle, ovarian morphology and hormonal parameters in rat models were examined. The rats exhibiting arrest in estrus phase following RU486 treatment represented the induction of polycystic ovary syndrome and were selected to observe the accumulation of the radiolabel particularly in the ovary. For this purpose 7.4 MBq of ^99m^Tc-noscapine was injected intravenously in the tail vein of PCOS rats and was compared with the same amount of activity in control rats. In addition it was also compared with the Tc-99m pertechnetate injected in both control and PCOS model.

### Gamma imaging studies

Scintigraphy was carried out after intravenous administration of the radiotracer (7.4 MBq) in the tail vein of female Wister rats and images were captured at 1, 2, 4 and 24 h post-administration using a dual head Hawkeye gamma camera system (GEMS, UK). All images were analyzed with in-built software Entegra Version-2. Animals were sedated by giving intramuscular injection of 0.75 ml/Kg body weight of calmpose and 1 mg/Kg body weight of ketamine throughout the experiment.

## Results

### Complexation studies

**Figure 1:**
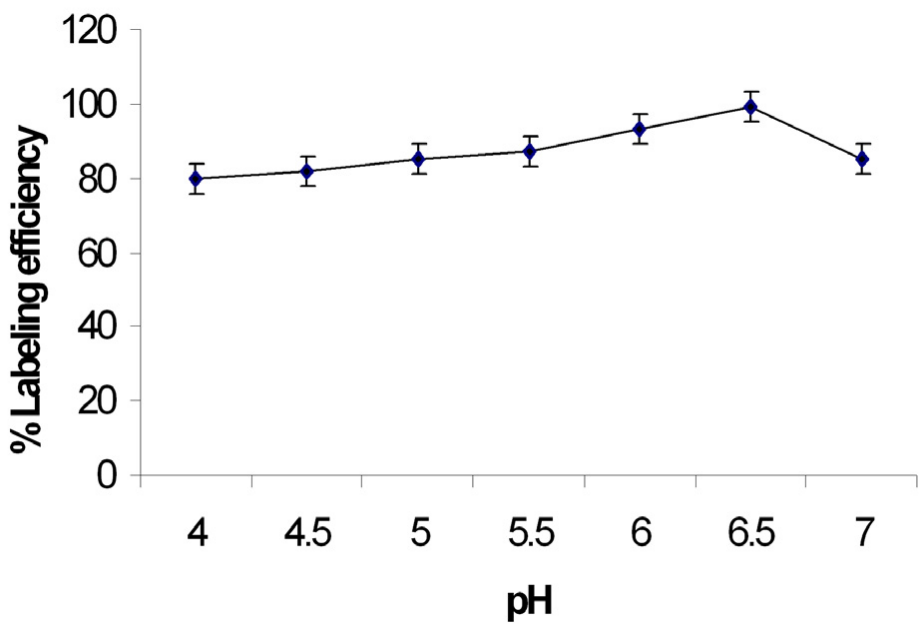
**Effect of pH on the stability of ^99m^Tc-noscapine**. Results are the mean of three separate experiments

On the basis of chromatographic analysis the radiolabeling efficiency was found to be more than 98% consistently. The optimal labeling efficiency was obtained with 50 μg of stannous chloride (the concentration of SnCl_2_.2H_2_O was varied from 10-400 μg) (Table 1) and at pH 6.5 (Figure 1).

**Table 1:** Effect of the concentration of stannous chloride dihydrate on the labeling efficiency of ^99m^Tc-noscapine.

SnCl_2_.2H_2_O concentration (μg/ml)	% Label	% R/H
10	86 ± 2.2	0.02 ± 0.01

20	92.5 ± 1.2	0.7 ± 0.04

50	98.9 ± 1.8	1.1 ± 0.1

100	90.9 ± 1.2	4.9 ± 0.1

200	88.0 ± 2.1	6.3 ± 0.4

400	82.0 ± 1.8	8.5 ± 0.1

### In vitro and in vivo stability studies

In vitro stability study showed that the labeled conjugate was fairly stable up to 24 h both in physiological saline (94.9% ± 2.0%) and serum (93.9% ± 1.8%) which correlated well with the in vivo stability studies (98.0% ± 2.4%) (Table 2).

**Table 2:** *In vitro and in vivo *stability studies of ^99m^Tc-noscapine.

Incubation Time (h)	Percentage Labeling *In Vitro*		Percentage Labeling *In Vivo*
	Saline	Serum	
0	98.9 ± 1.3	98.3 ± 1.5	98.6 ± 1.1
1	98.8 ± 1.2	98.6 ± 1.3	99.3 ± 1.4
2	99.0 ± 2.1	94.2 ± 1.5	99.2 ± 1.5
4	98.7 ± 1.2	94.1 ± 1.1	98.7 ± 1.0
6	96.0 ± 1.6	94.0 ± 1.5	98.6 ± 2.2
24	94.9 ± 2.0	93.9 ± 1.8	98.0 ± 2.4

Data is expressed as percentage of the total radioactivity in sample. Results are the mean of three separate experiments.

### Blood Clearance

**Figure 2:**
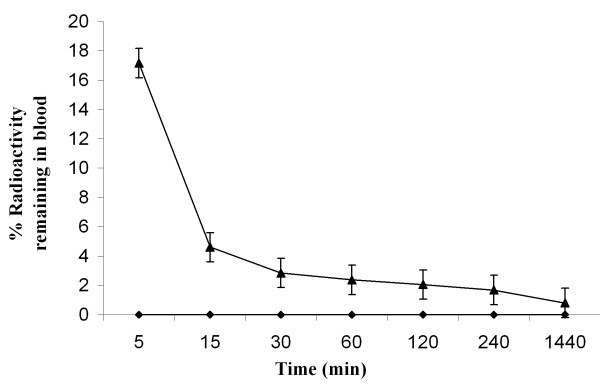
**Blood clearance of ^99m^Tc-noscapine (18.5 MBq) administered through ear vein in normal rabbit (n = 4)**.

*In vivo *clearance in rabbits revealed that there was a rapid wash out of the labeled drug from the circulation as 3% of the injected activity remained in the circulation at 1 h. After 1 h the clearance followed a slow pattern and at 24 h approximately 1.01% activity persisted in the blood (Figure 2). The biological half-life was found to be T_1/2_(Fast) ~12 minutes; T_1/2 _(Slow) 3 h and 50 minutes. The overall clearance of the radiolabeled molecule is consistent with known data of the parent molecule.

### Biodistribution of ^99m^Tc-noscapine in normal rats

Table 3 represents a comprehensive analysis of the compartmental organ distribution of ^99m^Tc-noscapine between 15 minutes to 24 h in healthy female rats. The study clearly indicates that the major route of excretion of radiopharmaceutical is hepatobiliary, since major accumulation was observed in liver than kidneys at 15 min (2.48 ± 0.78 ID/whole organ and 0.21 ± 0.13 ID/whole organ respectively). Spleen being an organ of high cell turnover also showed uptake 0.07 ± 0.005%ID/whole organ at 15 min which increased to 1.15 ± 0.75%ID/whole organ after 2 h post injection. In essence, negligible counts occurred in heart and brain but an appreciable activity was noticed in liver, kidney, ovary and urinary bladder. Specifically pronounced accumulation of the radiocomplex was observed in ovaries i.e. 0.09 ± 0.03%ID/whole organ at 1 h, 0.15 ± 0.03%ID/whole organ at 2 h and 0.53 ± 1.25% ID/whole organ at 4 h that reached 0.01 ± 0%ID/whole organ at 24 h post injection. The result is in concordance with the earlier reports that has shown noscapine localization in the aforementioned tissues and strengthens the fact that noscapine is behaving as noscapine when tagged with Tc-99m [18].

**Table 3:** Biodistribution of ^99m^Tc-noscapine in Wister rats following i.v. injection.

ORGAN	PERCENT INJECTED DOSE/WHOLE ORGAN (± SEM)				
	TIME				
	15 min	1 h	2 h	4 h	24 h
**Blood**	0.22 ± .04	0.116 ± .05	0.156 ± .05	0.115 ± .03	0.0085 ± .002
**Heart**	0.048 ± .007	0.023 ± .01	0.019 ± 2.35	0.015 ± .007	0.002 ± .001
**Liver**	2.48 ± .78	2.05 ± .52	1.36 ± 1.15	1.01 ± 1.37	0.135 ± .17
**Lungs**	0.28 ± .03	0.123 ± .11	0.21 ± .21	0.093 ± .06	0.002 ± .001
**Ovary**	0.123 ± .1	0.093 ± .03	0.15 ± .036	0.531 ± 1.25	0.01 ± 0
**Stomach**	0.088 ± .08	0.048 ± .07	0.043 ± .01	0.025 ± .007	0.002 ± .001
**Intestine**	0.11 ± .06	0.079 ± .07	0.063 ± .02	0.03 ± .01	0.003 ± 0
**Spleen**	0.07 ± .005	0.725 ± .85	1.156 ± .75	0.59 ± .79	0.137 ± .18
**Uterus**	0.076 ± .05	0.04 ± .01	0.03 ± .01	0.02 ± 0	0.004 ± 0
**Kidney**	0.21 ± .127	0.39 ± .23	0.44 ± .136	0.56 ± .16	0.088 ± .054
**Brain**	0.01 ± .006	0.005 ± .0025	0.006 ± .001	0.008 ± .001	0.001 ± 0

Data from five rats/group expressed as % injected dose/whole organ + SEM

### Preparation of PCOS Animal Model

**Figure 3:**
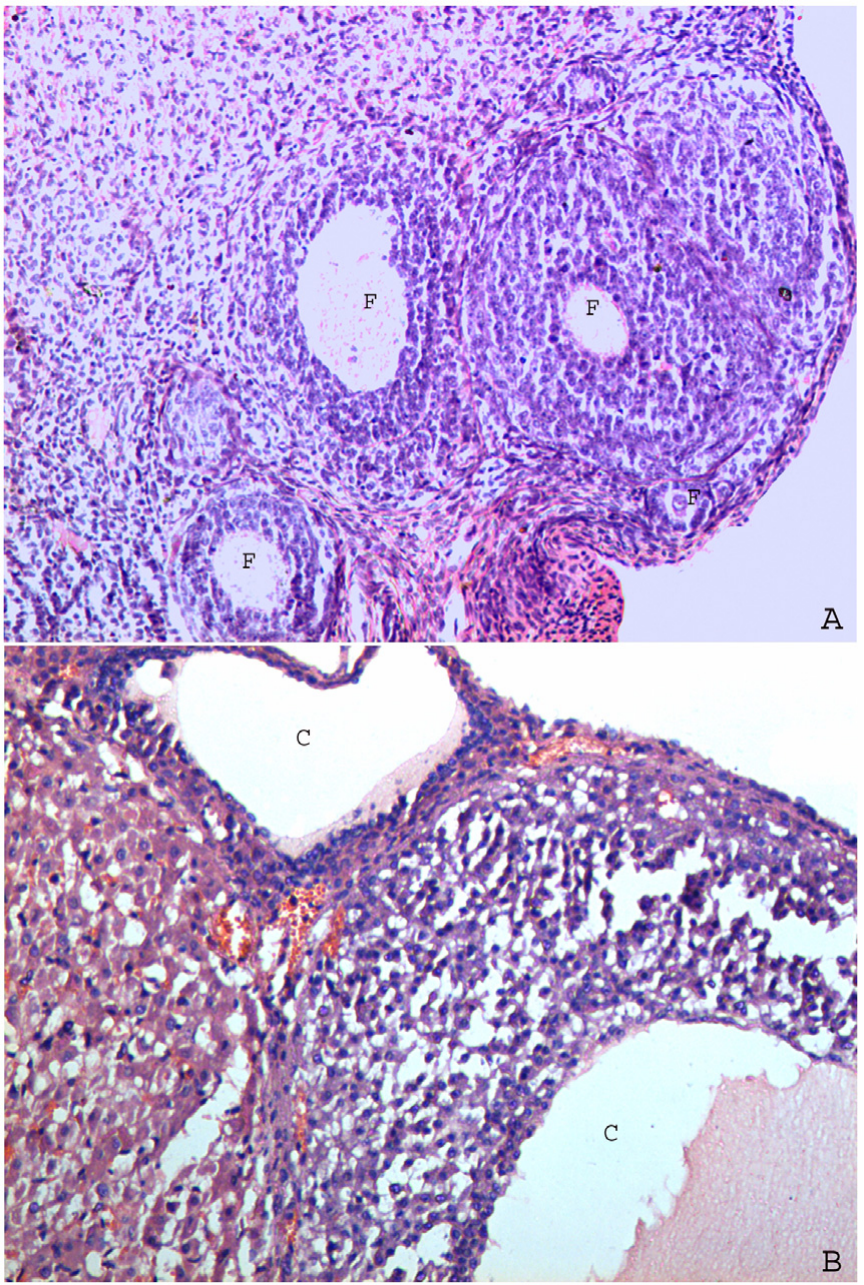
**Representative micrographs from the ovary of adult Wister rats treated with: olive oil, where F represents healthy follicles (A), RU486, where C represents follicular cyst formed due to hormonal imbalance (B)**.

Administration of antiprogesterone RU486 to 4-day-cyclic rats over 13 consecutive days starting on the day of estrus (day 1) induced an anovulatory cystic ovarian condition with endocrine and morphological features similar to those exhibited in polycystic ovarian disease (PCO) when compared to normal cyclic rats. Ovarian micrographs from control rats exhibited normal histology with healthy follicles (Figure 3A) whereas ovarian micrographs from PCOS induced rats showed abnormal cystic follicles with eroded granulose layer and thickened theca layer (Figure 3B).

### Gamma Scintigraphic imaging

**Figure 4:**
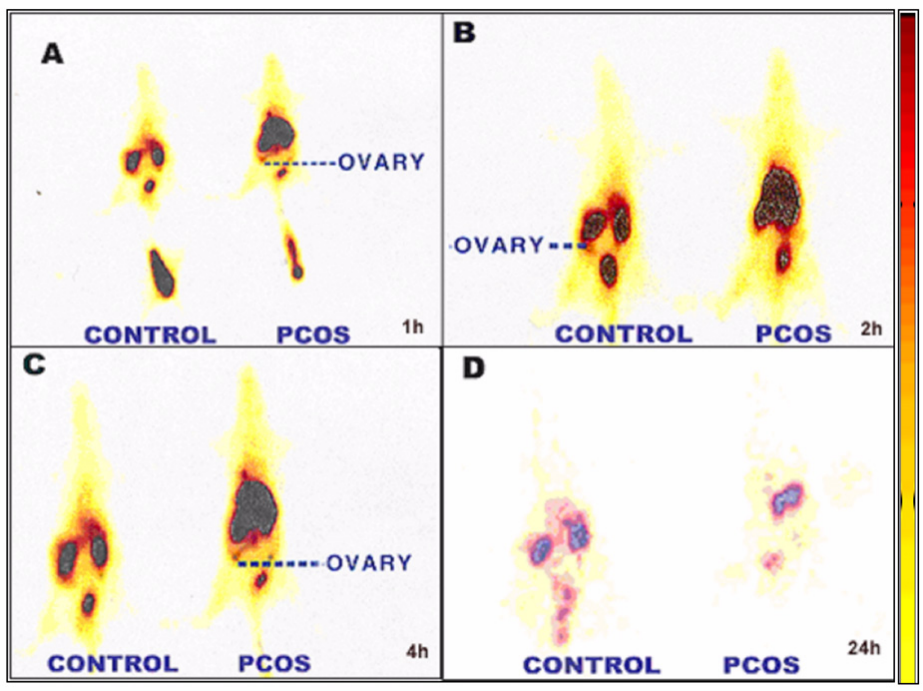
**Whole body scintigraphic images of ^99m^Tc-noscapine in control and PCOS induced female rats showing its accumulation in ovary at A: 1 h, B: 2 h, C: 4 h, D: 24 h**.

**Figure 5:**
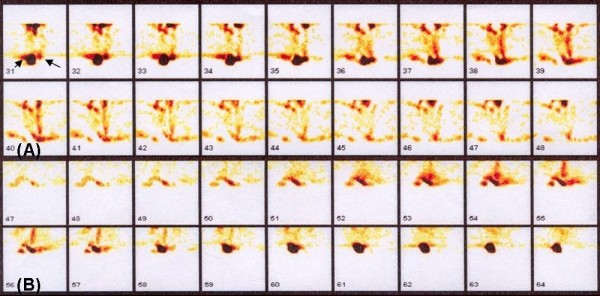
**Cut section coronal and transverse SPECT images of rat ovaries showing accumulation of ^99m^Tc-noscapine 2 h post administration**.

Localization of ^99m^Tc-noscapine in normal healthy rats and PCOS induced rats bearing cystic ovary over time, as determined by gamma camera imaging, is shown in Figure 4. The rats showed accumulation of activity in kidney and liver at 1 h, which reached to maximum at 4 h showing prominent uptake in ovary as well. Thus, the biodistribution pattern seen on non-invasive imaging with ^99m^Tc-noscapine was similar to the radiometric data obtained after sacrificing the animals. In a separate experiment (data not shown), rabbits imaged post ^99m^Tc-noscapine administration at different time intervals also showed accumulation of labeled complex in ovary, liver, kidney and skeletal tissue same as that observed in rats. Figure 5 shows the transverse and coronal cut section SPECT images of the radiotracer accumulation in rat ovaries at 2 hr post-injection.

### Specific uptake of ^99m^Tc-noscapine

Biodistribution studies in rats conducted to quantify localization of ^99m^Tc-noscapine in healthy and PCOS induced rats showed appreciable counts of ^99m^Tc-noscapine in ovary of PCOS induced rats (0.9 ± 0.03%) at 4 h as compared to normal rat ovary (0.53 ± 0.01%) (Table 4). Even at 2 h post injection, PCOS induced rats showed substantial localization of radioactivity in ovaries of PCOS induced rats (0.58 ± 0.05%) as compared to the control rats (0. 15 ± 0.005%). Tc-99m injected alone in PCOS induced rats showed negligible number of counts in ovaries at 2 h (0.05% ± 0.02%) and 4 h (0.06% ± 0.01%) respectively. This clearly reflects that noscapine is taken specifically by the ovaries as ^99m^Tc-noscapine shows pronounced accumulation in the ovaries in contrast to Tc-99m pertechnetate alone.

**Table 4:** A comparative analysis of ^99m^Tc-noscapine and ^99m^TcO_4_- uptake by control and PCOS induced rat ovary.

Time (h)	^99m^Tc-noscapine		^99m^TcO_4_-PCOS induced
	Normal Rat	PCOS induced	PCOS induced
2	0. 15 ± .005%	0.58 ± .05%**	0.05 ± .02%

4	0.53 ± .01%	0.9 ± .03%*	0.06 ± .01%

Each value is the mean ± standard deviation of data from five rats/group and the difference between the groups were tested using Student's t-test at the level *P < 0.05 and **P < 0.01.

## Discussion

Tc-99m pertechnetate, the non-specific control used in this study behaves chemically like sodium chloride. In PCOS induced rat ovaries, its accumulation was just 0.05% of the injected dose. In normal ovaries, its accumulation is known to be even lesser [30]. This accumulation in all probability represents the activity in blood pool and extracellular space, since pertechnetate is not known to internalize or interact specifically with the ovarian tissue. In contrast, the radiotracer uptake in normal ovary was 30 times higher as determined by radiometry, and more than 60 times in PCOS, with a rising pattern with time in both cases (p < 0.01). Literature for PCOS clearly suggests an interplay of hormonal and non-hormonal factors that influences microenvironment of ovary culminating in deranged hypothalamus-pituitary-ovarian (HPO) axis. Ovary and brain are among the tissues known to have noscapine receptors, which are hypothalamus specific [31,32] and could possibly affect the HPO axis. Significantly higher uptake of ^99m^Tc-noscapine in ovary as compared to simple technetium pertechnetate (TcO_4_^-^), strongly suggests receptor mediated specificity of the drug for ovarian tissue. Further increased ^99m^Tc-noscapine uptake seen in ovaries of PCOS induced animals, is probably in response to noscapine receptors which may be more predominant in PCOS tissue as compared to normal ovary. Compared to normal ovary, accumulation of ^99m^Tc-noscapine was 4 times higher at 2 h (p < 0.01) and 2 times higher at 4 h (p < 0.05) post-injection, again signifying the specific uptake and validity of PCOS model in studies involving noscapine or its radiolabeled form. Reduction in this specific uptake in ovary is consistent with normal behavior of noscapine which also shows relatively higher uptake in the brain (which contains noscapine receptors) in the initial phase only, showing complete washout within a few hours [31].

One of the major concerns in nuclear medicine research and radiopharmaceutical development is that the radiolabeled form of any drug should behave similar to the parent drug molecule. In case of noscapine too, apart from specific uptake at sites known to accumulate injected noscapine, there are a few other observations which confirm that ^99m^Tc-noscapine behaves substantially like the parent molecule. The blood clearance graph is typically biphasic in both cases with similar disappearance rates and other parameters [32,33] (Figure 2). The biological half-life of the radiopharmaceutical was found to be t_1/2 _(Fast) ≃ 12 minutes; t_1/2 _(Slow) ≃ 3 h and 50 minutes, while the reported t_1/2 _of the parent molecule is also 3 h (slow phase) [18]. Bioavailability is less than 1% of the injected dose at 4 h in both cases. The blood kinetic profile of radiolabeled noscapine showed its high target uptake with a diagnostically useful target-to non target ratio in a short period of time (Figure 3). Biodistribution study clearly indicates that the major route of excretion of the radiopharmaceutical is hepatobiliary. Spleen and bone marrow, being other organ/organ system with high cell turnover also showed significant uptake of Tc-99m noscapine (Table 3). In the absence of data on noscapine organ biodistribution in the world literature, it therefore appears that the biodistribution of ^99m^Tc-noscapine can be used as an effective guide, particularly in the early phase after intravenous administration. Later on, the bioequivalence is expected to become divergent due to fast metabolism of noscapine in the body [18,19].

Apart from radiometry data, gamma scintigraphy pattern suggests that ^99m^Tc-noscapine can probably be used as a specific radiotracer to study ovarian function and in imaging PCOS (Figure 4 and 5) and 2 h imaging is the optimum time for scintigraphy. SPECT images at 2 h post-injection further confirmed it to be the best protocol to image ovarian pathology. Fast initial clearance of ^99m^Tc-noscapine may be advantageous in this respect, giving good target-to-non target ratio (ovary Vs tissue background) in early phase of imaging. Planar and SPECT images show accumulation of the tracer particularly well in the diseased ovaries making ovary scintigraphy an exciting possibility. This preliminary observation is of value particularly because no radiopharmaceutical is available presently to image ovary or its dysfunction (PCOS). Further work involving interaction of ^99m^Tc-noscapine with in-vitro noscapine receptor models will strengthen this possibility. Dynamic biodistribution and imaging however suggest that the radiopharmaceutical may not be suitable for imaging brain noscapine receptors because of low initial uptake and early and fast washout.

In summary, the present study demonstrates a viable method to radiolabel noscapine with Tc-99m with high radiolabeling efficiency and stability along with its biodistribution and scintigraphic studies. Specific and high uptake of the radiotracer in ovary, particularly in case of PCOS suggests that ^99m^Tc-noscapine may have a diagnostic application in ovarian dysfunction.

## Competing interests

The authors declare that they have no competing interests.

## Authors' contributions

*AP *prepared the animal model for PCOS and executed animal experiments; *KC *was responsible for development and optimization of radiolabeling method for noscapine;* GM *executed scintigraphy experiments and co-wrote the paper;*AB *was responsible for conceptualization, macro- and microplanning, result analysis, and writing the paper. All authors participated in the discussion and interpretation of the final results, contributed to the final paper, and approved the final version submitted for publication.
